# The Coral Trait Database, a curated database of trait information for coral species from the global oceans

**DOI:** 10.1038/sdata.2016.17

**Published:** 2016-03-29

**Authors:** Joshua S. Madin, Kristen D. Anderson, Magnus Heide Andreasen, Tom C.L. Bridge, Stephen D. Cairns, Sean R. Connolly, Emily S. Darling, Marcela Diaz, Daniel S. Falster, Erik C. Franklin, Ruth D. Gates, Mia O. Hoogenboom, Danwei Huang, Sally A. Keith, Matthew A. Kosnik, Chao-Yang Kuo, Janice M. Lough, Catherine E. Lovelock, Osmar Luiz, Julieta Martinelli, Toni Mizerek, John M. Pandolfi, Xavier Pochon, Morgan S. Pratchett, Hollie M. Putnam, T. Edward Roberts, Michael Stat, Carden C. Wallace, Elizabeth Widman, Andrew H. Baird

**Affiliations:** 1 Department of Biological Sciences, Macquarie University, New South Wales 2109, Australia; 2 Australian Research Council Centre of Excellence for Coral Reef Studies, James Cook University, Townsville 4811, Australia; 3 Center for Macroecology, Evolution & Climate, Natural History Museum of Denmark, University of Copenhagen, Copenhagen DK-2100, Denmark; 4 Australian Institute of Marine Science, PMB #3, Townsville MC, Townsville 4810, Australia; 5 Department of Invertebrate Zoology, National Museum of Natural History, Smithsonian, Washington, District Of Columbia 20013, USA; 6 College of Marine and Environmental Sciences, James Cook University, Townsville 4811, Australia; 7 Marine Program, Wildlife Conservation Society, Bronx, New York 10460, USA; 8 University of Hawaii, Hawaii Institute of Marine Biology, School of Ocean and Earth Science and Technology, Kaneohe, Hawaii 96744, USA; 9 Department of Biological Sciences and Tropical Marine Science Institute, National University of Singapore, Singapore 117543, Singapore; 10 School of Biological Sciences, The University of Queensland, St Lucia, Queensland 4072, Australia; 11 Australian Research Council Centre of Excellence for Coral Reef Studies, School of Biological Sciences, The University of Queensland, St Lucia, Queensland 4072, Australia; 12 Environmental Technologies, Coastal & Freshwater Group, The Cawthron Institute, Nelson 7010, New Zealand; 13 Institute of Marine Science, The University of Auckland, Auckland 1142, New Zealand; 14 Trace and Environmental DNA Laboratory, Department of Environment and Agriculture, Curtin University, Perth, Western Australia 6102, Australia; 15 Biodiversity and Geosciences Program, Queensland Museum Network, South Brisbane, Queensland 4101, Australia; 16 School of Life Sciences, The University of Warwick, Coventry CV4 7AL, UK

**Keywords:** Community ecology, Marine biology, Biodiversity, Biogeography, Coral reefs

## Abstract

Trait-based approaches advance ecological and evolutionary research because traits provide a strong link to an organism’s function and fitness. Trait-based research might lead to a deeper understanding of the functions of, and services provided by, ecosystems, thereby improving management, which is vital in the current era of rapid environmental change. Coral reef scientists have long collected trait data for corals; however, these are difficult to access and often under-utilized in addressing large-scale questions. We present the Coral Trait Database initiative that aims to bring together physiological, morphological, ecological, phylogenetic and biogeographic trait information into a single repository. The database houses species- and individual-level data from published field and experimental studies alongside contextual data that provide important framing for analyses. In this data descriptor, we release data for 56 traits for 1547 species, and present a collaborative platform on which other trait data are being actively federated. Our overall goal is for the Coral Trait Database to become an open-source, community-led data clearinghouse that accelerates coral reef research.

## Background & Summary

Most ecosystems are rich in species that display a wide diversity of characteristics^1^ (i.e., traits). One way to make meaningful generalizations from this diversity has been to identify physiological, ecological or functional traits of organisms to infer (e.g., using traits as explanatory variables) patterns of demography, distribution and abundance, and more broadly, ecosystem function and evolution^2^. Moreover, species traits can be used as explanatory variables for the responses of ecosystems to environmental change, as functionally significant traits mediate species’ responses to disturbances^3^. Recently, research has demonstrated the utility of trait-based approaches for understanding the effects of anthropogenic disturbances^4^, the provisioning of ecosystem services^5^, species distributions^6–8^, species composition^9,10^, and energetic and ecological trade-offs^11,12^. In seminal papers, compilations of species trait data with broad taxonomic coverage have revealed, for example, a general axis of variation in plants that describes costs and benefits of key chemical, structural and physiological traits^11^; and factors influencing the metabolic rates of organisms^13^. However, such broad-scale insights have been restricted to relatively few taxonomic groups, often due to lack of data, particularly information about the ecological context in which data were collected, when such data do exist.

Trait data for stony corals (Cnidaria: Scleractinia) have been collected for more than 100 years and published in many languages. Sufficient data might well exist already for addressing broad-scale hypotheses regarding the ecology and evolution of corals. Although trait compilations are accumulating^4,14–16^, and new statistical approaches for analysing such data are emerging^7,12^, these datasets are typically gathered for specific traits in isolation to address specific questions which can result in duplication of effort by separate research groups (e.g., Darling *et al.*^12^ and Pratchett *et al.*^17^ both independently compiled growth rate data). Trait data also tend to be gathered rapidly, for instance with means extracted from tables that present a mixture of original data and data collected previously by others (i.e., meta-analyses). Such a rapid assembly of data can result in omission of important contextual information (e.g., local environmental conditions and levels of variation and replication), confusion about the origin of the data, preventing appropriate provenance and credit^18^, and the accidental duplication of data points in large datasets.

In this data descriptor, we introduce the Coral Trait Database: a curated database of trait information for coral species from the global oceans. The goals of the Coral Trait Database are: (i) to assemble disparate information on coral traits, (ii) to provide unrestricted, open-source access to coral trait data, (iii) to facilitate and encourage the appropriate crediting of original data sources, and (iv) to engage the reef coral research community in the collection and quality control of trait data. We release 56 error-checked, validated and referenced traits, and also provide their context of measurement, together with an online system for transparently and accurately archiving and presenting coral trait data in future research. Our vision is an inclusive and accessible data resource to more rapidly advance the science and management of a sensitive ecosystem at a time of unprecedented environmental change.

## Methods

The data are held in the Coral Traits Database (https://coraltraits.org). The database was designed to contain individual-level traits and species-level characteristics and is currently focused on shallow water zooxanthellate (‘reef building’) scleractinian corals. Individual-level traits include any potentially heritable quality of an organism^19,20^. In the database, individual-level traits are accompanied by contextual characteristics, which give information about the environment or situation in which an individual-level trait was measured (e.g., characteristics of the habitat, seawater or an experiment). These contextual variables are important for understanding variation in individual-level traits (e.g., as predictor variables in analyses). For example, if measurement of colony growth rate was measured at a given depth, the latter datum is included to provide important information for the focal measurement. Some individual-level traits have no or little variation (e.g., mode of larval development), and therefore contextual information is not required. Species-level characteristics do not have contextual information because they are characteristics of species as entities (such as geographical range size and maximum depth observed).

For simplicity, we use the single term ‘trait’ to refer to individual-level (variant and invariant), species-level (emergent) and contextual (environmental or situational) measurements. Moreover, these traits are grouped into ten use-classes based on various sub-disciplines of reef coral research: biomechanical, conservation, ecological, geographical, morphological, phylogenetic, physiological, reproductive, stoichiometric, and contextual.

### Observation and measurements

**Figure 1:**
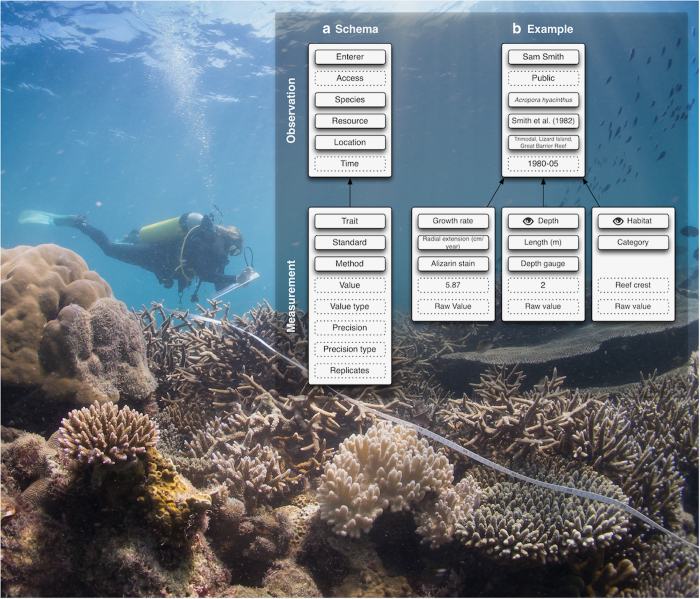
Overview of the design of the Coral Trait Database. (**a**) The general schema consists of an Observation of a coral colony that is a collection of one or more Measurements associated with the colony. Solid borders represent table associations and dotted borders represent values. Observations have four table associations (contributor, coral species, resource and location) and one value for access (i.e., public or private). Measurements have four table associations (observation, trait, methodology and standard) and five values. (**b**) An example of an observation where coral growth rate was measured along with two contextual measurements (represented in the database by an eye). All observation-level attributes are required. Required measurement-level attributes are trait, standard, value and value type. Precision details are entered when a value type is not a raw value. Photograph: Emily Darling.

The database contains two core data tables—Observations and Measurements—each of which has a series of associated tables (Fig. 1). We follow the high-level structure of the Observation and Measurement Ontology^21^ in that observations bind related measurements and potentially provide context for other observations.

The observation table contains information about the observation of a coral or coral species. Observation-level data must include the Enterer, Species, Location and Resource. Access is an optional variable, and can be controlled by database users entering data for a project that has not yet been published (see https://coraltraits.org/procedures for more information). Observation-level data are the same for all measurements corresponding to the observation. Measurement-level data include the Trait, Value, Standard (measurement unit), Methodology, and estimates of precision (if applicable). The hypothetical example given in Fig. 1b is for growth rate that was measured within the context of a water depth and habitat that were given in the published resource.

The Species table provides taxonomy that is regularly updated by the Taxonomy Advisory Board (https://coraltraits.org/procedures) to keep pace with the rapid rate of revision^22–24^. The table contains the valid name for each coral species based largely on the World Register of Marine Species (http://www.marinespecies.org), the major clade (Basal, Robust or Complex^25^), family based on molecular work^26^, family based on morphology (following Cairns^27^ or Veron^28^), and other names and synonyms.

### Data acquisition

**Figure 2:**
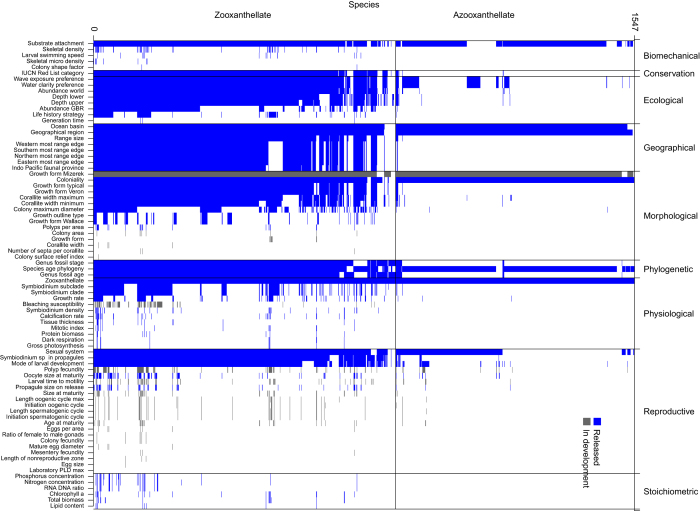
Trait by species matrix, illustrating coverage of trait data are currently available in the Coral Trait Database across the worlds 1547 coral species. Blue cells correspond with the traits released in this data descriptor. Grey cells correspond with other available data for which thorough error checking is still being conducted.

**Figure 3:**
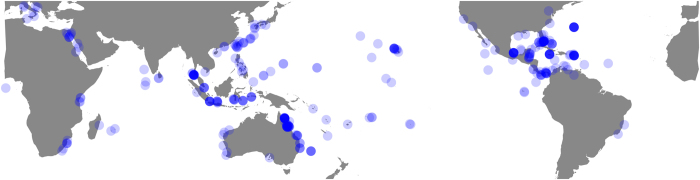


**Figure 4:**
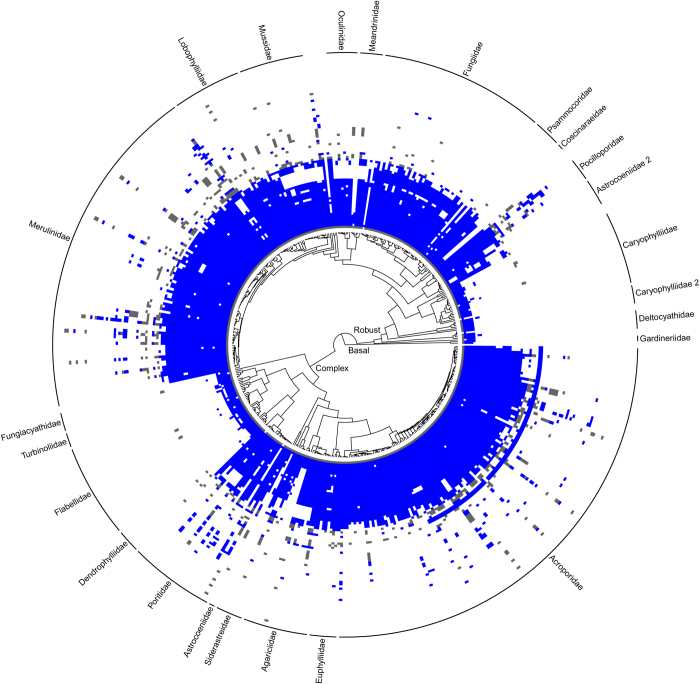
The phylogenetic coverage of traits in the Coral Trait Database, for the subset of species in the current molecular phylogeny. As for Fig. 2, blue cells indicate traits for species released in this data descriptor and grey cells indicate other available information in the database, still being federated.

All public data in the Coral Trait Database and included in this data descriptor release are linked with published resources, which include peer-reviewed papers, taxonomic monographs and books. The original source of entered data must be included (called the primary resource), even when extracted from secondary compilations (e.g., for the purpose of meta-analyses). Secondary sources can be included optionally, and so the database captures both the original data collector and subsequent data compilers, which allows both to be credited when re-using data. Measurement value types, which can be flexibly added to, currently include: raw, mean, median, maximum, minimum, expert opinion (the view of a single expert), group opinion (the consensus of a group of experts), and model derived. Continuous data are typically means extracted from tables or figures unless raw data are available. When available, aggregate values such as means and medians should be accompanied by the number of replicates and a measure of dispersion (e.g., standard deviation). Means and estimates of dispersion from figures in resources were captured using ImageJ^29^. The data released in this data descriptor have broad taxonomic (Fig. 2), global (Fig. 3) and phylogenetic (Fig. 4) coverage. However, some large data gaps exist, because few species have been comprehensively measured in many locations.

## Data Records

A static release of the 56 traits contained in this descriptor is available from the Coral Trait Database (Data Citation 1) and Figshare (Data Citation 2). Details and references for the trait data are summarised in Table 1 (available online only). Up-to-date data can be downloaded directly from the database. However, as validation (see Technical Validation, below) and data entry is ongoing, users are recommended to pull data from the static releases, to ensure results remain consistent as the database is updated. Both static releases and datasets downloaded from the database are accompanied by the primary (and, if applicable, secondary) resource lists for the data, which should be credited wherever feasible.

## Technical Validation

The database is curated on a voluntary basis, which includes a Managerial Board, Editorial Board, Taxonomy Advisory Board and Database Administrator (https://coraltraits.org/procedures). Database Contributors who add data for a new trait are typically asked to be that trait’s editor. Quality control of data and editorial procedures include:

1. **Contributor approval:** Database users must request permission to become a database contributor, and any observations entered by the contributor are associated with their user account.
2. **Editorial approval:** Once a contributor enters an observation of a coral trait, an email is sent automatically to the editor of that trait. The editor must approve the observation to remove the ‘pending’ flag from the observation record.
3. **User feedback:** Data issues can be reported for any observation using a simple form. Editors are automatically emailed if an issue with one of their traits is reported.
4. **Duplicate detection:** Measurements with the same value, resource, location and species are flagged for confirmation.
5. **Outlier detection:** Frequency histograms are generated in real time when loading trait pages. Outliers can be detected visually (e.g., a very large value for continuous data or a category that has one or few associated measurements for categorical data).

## Usage Notes

The data release is a compressed folder containing two files:

1. A csv-formatted data file containing all publicly available observation and measurement data, which includes contextual data.
2. A csv-formatted resource file containing all the resources (primary and secondary) that correspond with the data. Users are expected to cite the data correctly using these resources.

An example for extracting and reshaping release data for analysis can found online (https://coraltraits.org/procedures).

## Additional Information

Table 1 is only available in the online version of this paper.

**How to cite this article:** Madin, J. S. *et al.* The Coral Trait Database, a curated database of trait information for coral species from the global oceans. *Sci. Data* 3:160017 doi: 10.1038/sdata.2016.17 (2016).

## Supplementary Material



## Figures and Tables

**Table 1:** Overview of traits in release 1.1.1, including descriptions, measurement standards, the number of measurements and the references

Class	Name	Description	Standard	Default unit	Categories	Category descriptions	Measurements	References
Biomechanical	Colony shape factor	A dimensionless measure of mechanical vulnerability to hydrodynamic disturbance (see Madin and Connolly 2006). Colony shape factor is a function of colony size, and therefore each observation should also include a colony size measurement. Currently published data is only available for three species.	Dimensionless	N/A	N/A	N/A	1158	^30^
	Larval swimming speed	The swimming speed, typically the maximum, of coral larvae.	Speed	mm s^−1^	N/A	N/A	394	^31–40^
	Skeletal density	The material density of coral skeleton. Porosity measurements can be converted to density by multiplying the reciprocal of porosity by the maximum density of aragonite (2.94 g cm^−3).	Density	g cm^−3^	N/A	N/A	378	^16,17,41–74^
	Skeletal micro-density	The fine-scale specific gravity of the material from which coral skeleton is constructed (Bucher *et al.* 1998, following terminology from Barnes & Devereux 1988). Micro-density should be closer to the density of solid aragonite (~2.96 g cm^−3) than to typical bulk densities, because it does not capture corallite voids (i.e., porosity).	Density	g cm^−3^	N/A	N/A	9	^45^
	Substrate attachment	Whether or not individuals attach to substrates, including reef, rock and wood.	Category	N/A	attachedunattachedboth	Attached to the substratumNot attached to the substratumFound both attached and unattached to the substratum	1464	^28,75–77^
Conservation	IUCN Red List category	Red list categories are from Delbeek *et al.* (2009) as compiled by Carpenter *et al.* (2009).	Category	N/A	VULCNTDDCREN	VulnerableLeast concernNear threatenedData deficientCritically endangeredEndangered	818	^4,78^
Ecological	Abundance GBR	The typical local abundance of species when found on the Great Barrier Reef, Australia. Data were extracted from textual descriptions in Veron (1996) by Diaz and Madin (2011).	Category	N/A	rareuncommoncommon	Typically rare where foundTypically uncommon where foundTypically common where found	400	^79,80^
	Abundance world	The typical local abundance of species from Veron (2000). It is suspected that many species listed as rare are abundant at some localities. Furthermore, as Bridge *et al.* (2013) point out, some are abundant at depth.	Category	N/A	rareuncommoncommon	Typically rare where foundTypically uncommon where foundTypically common where found	823	^4,28,78,81–84^
	Depth lower	The maximum (deepest) observed depth of a species. Data are a mix of individual-level local observations and species-level global estimates based on expert opinion.	Length	m	N/A	N/A	1214	^4,78,85–105^
	Depth upper	The minimum (shallowest) observed depth of a species. Data are a mix of individual-level local observations and species-level global estimates based on expert opinion.	Length	m	N/A	N/A	1147	^4,78,87,89,95,97–99,102–105^
	Generation time	The average age of mothers in populations. This characteristic has only been empirically estimated for three species as far as we know ([Babcock 1991](/resources/273)). Values in Carpenter *et al.* (2008) are unreliable and we advise against using them.	Duration	years	N/A	N/A	3	^106^
	Life history strategy	Life history strategies broadly capture the various investments in growth, reproduction, and survivorship that differentiate species.	Category	cat	competitiveweedystress-tolerantgeneralist	Efficient at using resources and can dominate communities in productive environmentsOpportunistically colonize recently disturbed habitatsAdvantageous traits in chronically harsh environmentsDo well in habitats where competition is limited by low levels of stress and disturbance	143	^12^
	Water clarity preference	Preferred water clarity environment. Derived from preferred habitat textual descriptions, mostly from Veron and Stafford-Smith (2002), and published in Diaz and Madin (2011).	Category	N/A	bothclearturbid	Found in both clear and turbid water environmentsFound predominantly in clear water environmentsFound predominantly in turbid water environments	933	^28,79,81,83^
	Wave exposure preference	Preferred hydrodynamic exposure environment. Derived from preferred habitat textual descriptions, mostly from Veron and Stafford-Smith (2002), and published in Diaz and Madin (2011).	Category	N/A	protectedbroadexposed	Found predominantly in sheltered environmentsFound in both sheltered and exposed wave environmentsFound predominantly in exposed wave environments	933	^28,79,83^
Geographical	Eastern-most range edge	Eastern-most edge of a species range given as longitude, typically calculated from shapefiles. May also include one-off published observations. Not to be confused with eastern-most longitude relative to Greenwich. The value that results in the greatest range extent is used when species are synonymized.	Longitude	deg	N/A	N/A	709	^107^
	Geographical region	Presence in broad ocean and geographical regions.	Binomial	N/A	Indian OceanWestern and Central PacificWestern AtlanticEastern PacificEastern AtlanticSubantarctic and Antarctic	N/A	2316	^14,27^
	Indo-Pacific faunal province	Presence in the eleven Indo-Pacific faunal provinces established in Keith *et al.* (2013).	Category	N/A	Africa-IndiaAndaman-Nicobar IslandsAustralianFiji-Caroline IslandsHawaii-Line IslandsIndonesianJapan-VietnamPersian GulfPolynesiaRed SeaTonga-Samoa	N/A	3814	^7^
	Northern-most range edge	Northern-most edge of a species range given as latitude, typically calculated from shapefiles. May also include one-off published observations. The value that results in the greatest range extent is used when species have been synonymized.	Latitude	decimal degree	N/A	N/A	709	^107^
	Ocean basin	The ocean basin in which a species is found. Indian and Pacific Oceans are grouped as ‘pacific.’	Category	N/A	pacificatlantic	Present in the Indo-PacificPresent in the Atlantic	1494	^14,27^
	Range size	Geographic range size of species calculated from shapefiles. Be aware that there are different definitions of range size. For example, Veron (2000) range sizes are the sum of ecoregion sizes in which a species occurs; whereas, Hughes *et al.* (2013) range sizes capture the full extent of a species and so will be larger than Veron (2000) range sizes. Largest range size is used when species are synonymized.	Area	km^2^	N/A	N/A	1477	^28,107^
	Southern-most range edge	Southern-most edge of a species range given as latitude, typically calculated from shapefiles. May also include one-off published observations. The value that results in the greatest range extent is used when species have been synonymized.	Latitude	decimal degree	N/A	N/A	709	^107^
	Western-most range edge	Western-most edge of a species range given as longitude, typically calculated from shapefiles. May also include one-off published observations. Not to be confused with western-most longitude relative to Greenwich. The value that results in the greatest range extent is used when species have been synonymized.	Longitude	decimal degree	N/A	N/A	709	^107^
Morphological	Coloniality	Whether mature individuals of a species are colonial, solitary or either colonial or solitary (both).	Category	N/A	colonialsolitaryboth	Mature individuals are colonialMature individuals are solitaryMature individuals can be either colonial or solitary	1613	^28,77^
	Colony maximum diameter	The maximum diameter of a colony. At this stage, most maximum diameters have been extracted from monographs. However, new published records of large colonies should also be entered.	Length	cm	N/A	N/A	537	^28,75,80,83,84,108–128^
	Corallite width maximum	The maximum typical corallite width, axial corallite width or valley size.	Length	mm	N/A	N/A	733	^28,77,81,121,129–133^
	Corallite width minimum	The minimum typical corallite width, axial corallite width or valley size.	Length	mm	N/A	N/A	688	^28,77,81,121,129–133^
	Growth form typical	The growth form (morphology) of a species as derived from text descriptions in Veron (2000). The ‘typical’ growth form is given for each species, rather than all forms that might be observed in the field.	Category	N/A	encrustinglaminarsubmassivemassivecolumnarbranching_closedbranching_opentables_or_platesdigitatecorymbosehispidoseencrusting_long_uprights	Overlaying the substratumThin sheets often forming whorlsNot quite massiveSolid with similar shape in all directionForming columnsBranches in clusters or tuftsBranches of similar length given off at similar anglesColony outline in the shape of a table i.e., a top with one central leg or side-attached tableEncrusting with regular short upright branchesFlat topped clumpsOpen-branched except with a second type of branch given off at regular intervalsOverlaying the substratum with long branches	773	^28^
	Growth form Veron	The growth form (morphology) of a species as derived from text descriptions in Veron (2000). Species can have more than one growth form, and therefore captures some degree of morphological plasticity.	Category	N/A	encrustinglaminarsubmassivemassivecolumnarbranching_closedbranching_opentables_or_platesdigitatecorymbosehispidoseencrusting_long_uprights	Overlaying the substratumThin sheets often forming whorlsNot quite massiveSolid with similar shape in all directionForming columnsBranches in clusters or tuftsBranches of similar length given off at similar anglesColony outline in the shape of a table i.e., a top with one central leg or side-attached tableEncrusting with regular short upright branchesFlat topped clumpsOpen-branched except with a second type of branch given off at regular intervalsOverlaying the substratum with long branches	1168	^28^
	Growth form Wallace	The growth form (morphology) of a species as derived from text descriptions in Wallace (2012). Species may, but tend not to, have more than one growth form.	Category	N/A	arborescentarborescent_tablescorymbosecaespitosecaespitose_corymbosehispidoseencrustingelkhorncuneiformtables_or_plates	Branches of similar length given off at a similar angles. Open branchingOpen branched tablesFlat topped clumpsBranches in clusters or tufts. Closed branchingFlat topped closed branching clumpsArborescent except with a second type of branch given off at regular intervals around the primary branchAdhering to or overlaying the substratumBranches in the shape of the horns of an ElkBranches shaped like a wedgeColony outline in the shape of a table, i.e., a top with one central leg or pedicle, may be side-attached table	122	^77^
	Growth outline type	Whether or not a colony tends to approach a predictable outline. This trait was included in Wallace *et al.* (2012), and so has been measured mostly for Acropora.	Category	N/A	Indeterminatedeterminate	Colony grows apparently without any intrinsic restrictionColony grows to a more or less predictable outline	119	^77^
	Polyps per area	The number of polyps found in a given colony surface area.	Density	units cm^−2^	N/A	N/A	55	^16,70,72,106,121,129,131–147^
Phylogenetic	Genus fossil age	Date of the first palaeontological occurrence of morphologically defined genera based on the published literature.	Million years ago	mya	N/A	N/A	3799	^7,28,148–150^
	Genus fossil stage	The geochronological unit of the first palaeontological occurrence of morphologically defined genera based on the published literature.	Category	N/A	Recent, Eocene, Oligocene, Miocene, Ypresian, Miocene middle, Cretaceous Lower, Aptian, Jurassic Upper, Eocene middle, Cretaceous Upper, Turonian, Cretaceous, Cretaceous upper, Pleistocene, Priabonian, Cretaceous middle, Pliocene, Barremian, Neocomian, Chattian, Thanetian, Danian, Kimmeridgian, Miocene upper, Burdigalian, Oligocene middle, Rupelian upper, Tortonian, Cenomanian, Pleistocene-?Oligocene, Miocene Lower, Aquitanian, Eocene-Cretaceous, Pliocene-Pleistocene, Palaeocene, Rupelian, Bathonian	N/A	2335	^28,149,150^
	Species age phylogeny	This is the phylogenetic tip length based on a phylogeny of 1547 species reconstructed using supertree and MCMC methods, incorporating molecular, morphological and taxonomic data.	Million years ago	mya	N/A	N/A	1461	^151^
Physiological	Calcification rate	The rate at which aragonite is laid down per unit of skeletal surface area. When using this data, be aware that this trait is measured in numerous ways.	Percent per year	% yr^−1^	N/A	N/A	320	^16,17,41,43,46,48,50,53,54,63,67,70–72,74,152–170^
	Dark respiration	The rate of oxygen consumption measured in the darkness per unit of skeletal surface area. Values may include both light enhanced dark respiration and dark acclimated dark respiration.	Rate	μmol O_2_ cm^−2^ h^−1^	N/A	N/A	46	^16,138,139,144,152,157,171–179^
	Gross photosynthesis	The rate of oxygen production measured in the light per unit of skeletal surface area. This includes oxygen consumption due to light respiration.	Rate	μmol O_2_ cm^−2^ h^−1^	N/A	N/A	37	^16,138,139,144,152,157,171–173,175–179^
	Growth rate	Typically, the yearly extension for branching and massive corals, or simple linear extension. Growth rate is sometimes measured using different dimensions (e.g., diameter and radius) or over shorter periods of time (e.g., month), which are indicated by measurement standards and methodologies, and so values may need to be standardised before comparisons among measurements can be made.	Extension rate (linear)	mm yr^−1^	N/A	N/A	1297	^12,16,17,41,43,46,48,50,51,53,54,56,57,59,61–63,65–68,70–74,106,113,138,169,170,180–316^
	Mitotic index	The percentage of cells in the paired stage of cell division.	Percent	%	N/A	N/A	31	^16,317–322^
	Protein biomass	The amount or biomass of protein per unit of skeletal surface area.	Density	mg cm^−2^	N/A	N/A	32	^16,138,139,171,179,323–329^
	Symbiodinium clade	The genetic identity of Symbiodinium found in coral tissue at the clade level (broad level of major symbiont taxa). This is typically identified using regions of the nuclear ribosomal DNA, but other regions are also used.	Category	N/A	A, B, C, D, F, G, H, I	N/A	3147	^15,330–379^
	Symbiodinium density	The number of symbiont cells per unit of skeletal surface area.	Density	units cm^−2^	N/A	N/A	4062	^16,164,171,320,380–393^
	Symbiodinium subclade	The genetic identity of Symbiodinium found in coral tissue at the level below clade, but usually above species. This is typically identified using the nuclear ribosomal DNA Internal Transcribed Spacer region (ITS2), but other markers are also used.	Category	N/A	N/A	N/A	3068	^15,76,330–338,340–345,347–365,367–379^
	Tissue thickness	The distance from the external surface to the internal surface of the coral tissue.	Length	mm	N/A	N/A	59	^16,72,273,306,388,394^
	Zooxanthellate	Is the species zooxanthellate?	Category	N/A	zooxanthellateazooxanthellateboth	Contain zooxanthellae within their tissuesDon't contain zooxanthellae within their tissuesSometimes contain zooxanthellae within their tissues	1548	^27,28,75,76,78,91,395–420^
Reproductive	Mode of larval development	The mode of larval development classified as either a brooder, where fertilization is internal and colonies release planulae larvae, or a broadcast spawner, where gametes are release for external fertilization and the planulae develops in the plankton.	Category	N/A	bothbrooderspawner	individual colonies both brood and spawnFertilization internalFertilization external	814	^14,32,35,84,120,141,146,234,235,421–523^
	Oocyte size at maturity	The diameter of mature oocytes in a population. Determined by histology or dissection or measuring the size of eggs once released from the colony in broadcast spawners.	Length	μm	N/A	N/A	133	^234,423,425,428,436,441,448,460,478–480,483,484,493,494,498,500,510,519,521,524–530^
	Propagule size on release	The size of eggs or planula larvae on release.	Length	μm	N/A	N/A	67	^423,425,428,478,480,483,484,493,510,512,525,528–531^
	Sexual system	Each polyp of the population having gametes of only one sex (either male or female) at maturity (gonochore); one or more polyps of the population having both male and female gametes at maturity (hermaphrodite).	Category	N/A	gonochorehermaphrodite	Only one sex in all polypsBoth sexes in at least one polyp	1153	^14,35,84,141,146,234,235,422–429,432,436,438–443,445–449,451–454,456–458,460,461,463–469,471,472,476–481,483–489,492–513,515–523,525,532–550^
	Symbiodinium sp. in propagules	Whether or not mature eggs or larvae contain Symbiodinium sp. at the time of release from the parent. Typically determined by eye, rarely by histology or fluorescent microscopy, which are required for confirmation.	Binomial	N/A	yesno	Symbiodinium sp. in propagulesNo Symbiodinium sp. in propagules	818	^14,120,146,234,235,424–427,429,436,438,440,441,444,447,451,453,455,456,458,460,466,468,473,474,476,479,483,484,488,489,493–501,505,511,512,517,522,525,535,538,541,549,551–555^
Stoichiometric	Chlorophyll a	The amount of chlorophyll a in coral tissue, typically given per unit surface area.	Density	μg cm^−2^	N/A	N/A	110	^16,144,171,179,215,222,323,328,329,383,388,389,556–571^
	Lipid content	The amount of lipid is tissue.	Density	mg cm^−2^	N/A	N/A	13	^16,325–328,385,572^
	Nitrogen concentration	The amount of nitrogen in tissue.	Percent	%	N/A	N/A	131	^573^
	Phosphorus concentration	The amount of phosphorus in tissue.	Percent	%	N/A	N/A	142	^573^
	RNA:DNA ratio	The relative quantities of RNA and DNA.	Ratio	x:y	N/A	N/A	80	^573^
	Total biomass	The dry weight of holobiont tissue, typically reported as mass per unit of skeletal surface area of a colony.	Density	mg cm^−2^	N/A	N/A	3867	^16,138,139,157,325,327,328,389,392,560,564,574,575^
N/A denotes not applicable.								
