# Corrigendum: The Coral Trait Database, a curated database of trait information for coral species from the global oceans

**DOI:** 10.1038/sdata.2017.174

**Published:** 2017-12-05

**Authors:** Joshua S. Madin, Kristen D. Anderson, Magnus Heide Andreasen, Tom C.L. Bridge, Stephen D. Cairns, Sean R. Connolly, Emily S. Darling, Marcela Diaz, Daniel S. Falster, Erik C. Franklin, Ruth D. Gates, Aaron M.T. Harmer, Mia O. Hoogenboom, Danwei Huang, Sally A. Keith, Matthew A. Kosnik, Chao-Yang Kuo, Janice M. Lough, Catherine E. Lovelock, Osmar Luiz, Julieta Martinelli, Toni Mizerek, John M. Pandolfi, Xavier Pochon, Morgan S. Pratchett, Hollie M. Putnam, T. Edward Roberts, Michael Stat, Carden C. Wallace, Elizabeth Widman, Andrew H. Baird

*Scientific Data* 3:160017 doi: 10.1038/sdata.2016.17 (2016); Published 29 March 2016; Updated 5 December 2017.

The authors regret that Aaron Harmer was omitted in error from the author list of the original version of this Data Descriptor. This omission has now been corrected in the HTML and PDF versions.

**Contributions**

J.S.M. and A.H.B. conceived the idea. J.S.M., M.D. and A.H.B. compiled the preliminary data. J.S.M., K.D.A., A.H.A., T.B., S.D.C., S.C., S.R.C., E.D., M.D., D.F., E.C.F., R.D.G., A.M.T.H., M.O.H., D.H., S.A.K., M.A.K., C.K., J.M.L., C.E.L., O.L., J.M., T.M., J.M.P., X.P., M.S.P., H.M.P., T.E.R., M.S., C.C.W., E.W. and A.H.B. compiled, entered and edited trait data and jointly wrote the data descriptor.

